# Rationale and design of oBservational clinical Research In chronic kidney disease patients with renal anemia: renal proGnosis in patients with Hyporesponsive anemia To Erythropoiesis-stimulating agents, darbepoetiN alfa (BRIGHTEN Trial)

**DOI:** 10.1007/s10157-017-1427-4

**Published:** 2017-06-28

**Authors:** Hideki Kato, Masaomi Nangaku, Hideki Hirakata, Takashi Wada, Terumasa Hayashi, Hiroshi Sato, Yasushi Yamazaki, Takao Masaki, Tatsuo Kagimura, Hiroyasu Yamamoto, Hiroki Hase, Masahiro Kamouchi, Enyu Imai, Kyoichi Mizuno, Manabu Iwasaki, Tadao Akizawa, Yoshiharu Tsubakihara, Shoichi Maruyama, Ichiei Narita

**Affiliations:** 1 Division of Nephrology and Endocrinology, The University of Tokyo Graduate School of Medicine, 7-3-1 Hongo, Bunkyo, Tokyo, 113-8655 Japan; 2 Fukuoka Renal Clinic, Fukuoka, Fukuoka Japan; 3 Department of Nephrology and Laboratory Medicine, Faculty of Medicine, Institute of Medical, Pharmaceutical and Health Sciences, Kanazawa University, Kanazawa, Ishikawa Japan; 4 Department of Kidney Disease and Hypertension, Osaka General Medical Centre, Sumiyoshi, Osaka, Japan; 5 Division of Clinical Pharmacology and Therapeutics, Tohoku University Graduate School of Pharmaceutical Sciences and Faculty of Pharmaceutical Sciences, Sendai, Miyagi Japan; 6 Department of Nephrology and Rheumatology, Kagawa Prefectural Central Hospital, Takamatsu, Kagawa Japan; 7 Department of Nephrology, Hiroshima University Hospital, Hiroshima, Hiroshima Japan; 8 Translational Research Informatics Center, Foundation Biomedical Research and Innovation, Kobe, Hyogo Japan; 9 Department of Internal Medicine, Atsugi City Hospital, Atsugi, Kanagawa Japan; 10 Division of Nephrology, Toho University Ohashi Medical Center, Meguro, Tokyo Japan; 11 Department of Health Care Administration and Management, Graduate School of Medical Sciences, Kyushu University, Fukuoka, Fukuoka Japan; 12 Nakayamadera Imai Clinic, Takarazuka, Hyogo, Department of Nephrology, Fujita Health University Toyoake, Toyoake, Aichi Japan; 13 Mitsukoshi Health and Welfare Foundation, Shinjuku, Tokyo, Japan; 14 Department of Computer and Information Science, Seikei University, Musashino, Tokyo, Japan; 15 Division of Nephrology, Department of Medicine, Showa University School of Medicine, Shinagawa, Tokyo, Japan; 16 Course of Safety Management in Health Care Sciences, Graduate School of Health Care Sciences, Jikei Institute, Yodogawa, Osaka, Japan; 17 Department of Nephrology, Nagoya University Graduate School of Medicine, Nagoya, Aichi Japan; 18 Division of Clinical Nephrology and Rheumatology, Niigata University Graduate School of Medical and Dental Sciences, Niigata, Niigata Japan

**Keywords:** Darbepoetin alfa, Erythropoiesis-stimulating agent, Hemoglobin, Chronic kidney disease, Erythropoietin resistance

## Abstract

**Background:**

Renal anemia is an important complication in non-dialysis chronic kidney disease (CKD) patients as well as in dialysis patients. Although recombinant human erythropoietin has dramatically improved prognosis and quality of life in these patients, there have been issues among non-dialysis CKD patients who exhibit hyporesponsiveness to erythropoiesis-stimulating agent (ESA). The causes and definition of ESA hyporesponsiveness, as well as the incidence of renal and cardiovascular disease (CVD) events in such patients, are yet to be clarified.

**Methods:**

This ongoing trial is a multicenter, prospective, observational study of non-dialysis CKD patients with renal anemia. The primary objective is to survey the current realities of the therapy with ESA in Japan and evaluate the correlation between hyporesponsiveness to darbepoetin alfa and CKD progression. The secondary objective is to investigate relationship between ESA hyporesponsiveness and CVD events based on the clinical situation in Japan, and to explore an ESA response index.

**Results:**

The subjects consist of CKD patients with estimated glomerular filtration rate (eGFR) below 60 mL/min/1.73 m^2^ who present renal anemia. The target number of registered cases is 2000 patients, based on estimates of incidences of renal and CVD events from past studies. Renal function and CVD events will be observed for 96 weeks after the initiation of darbepoetin alfa administration. Definitions of ESA hyporesponsiveness will also be investigated.

**Conclusion:**

By clarifying markers and factors involved in ESA hyporesponsiveness and their relationships with renal and CVD events, this ongoing study aims to improve evidence-based therapies for renal anemia in non-dialysis CKD patients.

## Introduction

Chronic kidney disease (CKD) is a common health disorder worldwide, and its age-adjusted mortality rate has grown annually [1]. CKD is the eighth leading cause of death, affecting an estimated 13% of adults in Japan [2–4]. CKD is defined as the presence of an estimated or measured glomerular filtration rate less than 60 mL/min/1.73 m^2^ and/or evidence of kidney damage which last for at least 3 months.

Renal anemia is treated using recombinant human erythropoietin (rHuEPO) or other erythropoiesis-stimulating agent (ESA). The indications for ESAs have been expanded beyond dialysis patients to non-dialysis CKD patients, and these drugs have been reported to have variety of beneficial effects, including improvement in quality of life, preservation of renal functions [5, 6], a significant decrease in blood transfusion requirement, and improvement in the prognosis of non-dialysis and dialysis patients [4].

Recently, attention has been paid to patients who exhibit hyporesponsiveness to ESA, in which hemoglobin (Hb) concentration does not increase significantly despite ESA administration. The major cause of ESA hyporesponsiveness is iron deficiency, and other prominent factors are considered hemorrhage, generalized disorder of hematopoiesis (infection, inflammation, folic acid, or vitamin B12 deficiency), hematologic malignancies including myelodysplastic syndrome, hypersplenism, antibody-mediated pure red-cell aplasia (PRCA), and malnutrition [7]. However, at present, we still do not have a clear definition of ESA hyporesponsiveness.

The “Guidelines for Renal Anemia in Chronic Kidney Disease” published in Japan in 2016 state that “the lack of a standard method for evaluating ESA responsiveness make it difficult to reach clear definitions of ESA hyporesponsiveness and high-dose ESA” [4]. Neither do the Kidney Disease Improving Global Outcomes (KDIGO) guidelines provide a definition, stating that ESA hyporesponsiveness is defined if they have no increase in Hb concentration from baseline after the first month of ESA treatment on appropriate weight-based dosing [7]. In this context, there is a need for the definition and evidence-based markers of ESA hyporesponsiveness and clinical guidelines for anemia management in ESA hyporesponsive patients.

The results of large clinical trials on ESA therapy for renal anemia in non-dialysis CKD patients have been reported. These include the CHIOR trial [8] and the TREAT trial [9], which examined mortality and CVD events at a target Hb concentration, the former using epoetin alfa and the latter using darbepoetin alfa. In these trials, groups using ESA with a higher target Hb concentration set near the normal value of healthy adults (13.0 g/dL or higher) exhibited a higher incidence of CVD events, suggesting the possibility that although ESA may normalize Hb concentration, it could also increase the risk of CVD incidence. However, subsequent sub-analysis of CHIOR trial found that regardless of the target Hb concentration applied, patients who achieved the target Hb concentration had better outcome than those who did not [10]. Moreover, a sub-analysis of the TREAT trial reported in 2010 found that an ESA hyporesponsive group exhibited a lower mean Hb concentration, greater incidence of a composite CVD endpoint, and higher mortality, despite being administered large doses of ESA [11]. In other words, the complications may not be attributed simply to the elevated Hb concentrations, but patients who exhibit ESA hyporesponsiveness may have poor prognoses, or that administering high-dose ESA itself results in deteriorated outcomes. The above results demonstrate the importance of examining ESA responsiveness as well as the target Hb concentration when undergoing ESA therapy in non-dialysis CKD patients. Further trials are necessary to verify these problems.

In this ongoing trial, we will perform a multicenter, prospective, observational study of non-dialysis CKD patients with renal anemia.

The primary outcome is to survey the current realities of the therapy with ESA in Japan and evaluate the factors and correlation between hyporesponsiveness to darbepoetin alfa and CKD progression based on the clinical situation in Japan. The secondary outcome is to investigate the relationship between ESA hyporesponsiveness and CVD events. By analyzing our data, we will also establish the definition of the hyporesponsiveness to ESA and ESA responsive index (ERI).

## Methods

### Trial design

“oBservational clinical Research In chronic kidney disease patients with renal anemia: renal proGnosis in patients with Hyporesponsive anemia To Erythropoiesis-stimulating agents, darbepoetiN alfa (BRIGHTEN Trial)” established the Steering Committee, the Data Coordinating Center, as well as the Event Assessment Panel to survey the current realities of the therapy with ESA in Japan and factors related to hyporesponsiveness to darbepoetin alfa and CKD progression. This is an ongoing multicenter, prospective, and observational study of non-dialysis CKD patients with renal anemia who are scheduled to have treatment with darbepoetin alfa. Since this is observational study, we do not intervene the therapy of renal anemia nor assign the patients. We started the enrollment in May 2014 after the approval of the protocol by the institutional review board (IRB) at the main institution.

Besides darbepoetin alfa, several other long-acting ESAs are currently used in Japan. However, since the pharmacodynamics and effects of these drugs vary widely, and this drug was used in the TREAT trial [9] which is the leading source for evidence on ESA hyporesponsiveness, the ESA used in the present trial was limited to darbepoetin alfa. The study was registered to ClinicalTrials.gov (NCT02136563) and UMIN-CTR (UMIN000013464). The study protocol and informed consent form were approved by the institutional review board of each center. All patients received information regarding the purpose and nature of this study as well as the potential risks and benefits. Written informed consent was obtained from all individual participants included in the study. This study is being conducted under the health insurance system of Japan, in accordance with the 1964 Declaration of Helsinki and its later amendments, and the Ethical Guidelines on Clinical Studies of the Ministry of Health, Labor and Welfare of Japan.

### Participants

The following describe the definition of the diseases, eligibility criteria of the CKD and renal anemia, and the exclusion criteria for this trial.

### Definition of CKD [3]

The presence of (1), (2), or both of the following criteria which last for at least 3 months:

1. The presence of kidney damage from blood or urine, imaging, or pathological abnormalities.
2. GFR <60 mL/min/1.73 m^2^. Estimated GFR (eGFR) is calculated using the equation for Japanese population [12].

### Definition of renal anemia [4]

1. Anemia caused by insufficient EPO production in the kidneys due to CKD.
2. Anemia due to impaired erythropoiesis, decreased erythrocyte life span, iron metabolism dysfunction, nutritional disorders, and other factors observed in CKD.

### Eligibility criteria

Non-dialysis CKD patients with renal anemia, who fulfilled all the inclusion criteria, do not fall under any of the exclusion criteria at registration.

### Inclusion criteria

1. Patients scheduled to have treatment with darbepoetin alfa for the first time within 8 weeks after registration.
2. Patients with eGFR less than 60 mL/min/1.73 m^2^ at the most recent examinations up to 8 weeks prior to registration.
3. Patients with Hb concentration less than 11.0 g/dL at the most recent examination up to 8 weeks prior to registration.
4. Patients aged more than 20 years when consent was obtained.
5. Patients who provided written informed consent.

### Exclusion criteria

1. Patients scheduled to initiate maintenance dialysis or undergo kidney transplantation up to 24 weeks after registration.
2. Patients with a history of ESA treatment. Those administered ESA temporarily more than 12 weeks before registration are eligible.
3. Patients with malignant tumors, hematologic diseases, or hemorrhagic diseases.
4. Patients hypersensitive to ESA or its ingredients.
5. Patients who are pregnant or possibly pregnant, breast feeding, or wish to become pregnant during the trial.
6. Patients participating in other clinical trials.
7. Patients deemed unsuitable by the investigator of this trial.

### Treatment

The subjects include non-dialysis CKD patients diagnosed with renal anemia. Patients who are identified as the indication of ESA treatment and who consent to participate in this study are registered. Administration of darbepoetin alfa is initiated within 8 weeks after registration. Darbepoetin alfa is administered according to the package insert, and patients are observed for 96 weeks from the administration. Treatment will be continued if necessary after the observation period ends. The following methods, based on guidelines and the package insert, are recommended for administering darbepoetin alfa.

In addition, iron supplement is administered according to the guidelines of renal anemia in Table 1 without restriction.

**Table 1:** Indication of Hb concentration to initiate ESA and target Hb concentration based on different guidelines [3, 4, 7]

Guidelines	Indication to initiate ESA	Target Hb concentration
The Japanese Society for Dialysis Therapy 2008	Less than 11 g/dL	11 g/dL or higher. If Hb exceeds 13 g/dL, consider dose reduction or suspension
The Japanese Society for Dialysis Therapy 2015	Less than 11 g/dL	11 g/dL or higher. If Hb exceeds 13 g/dL, consider dose reduction or suspension
CKD Clinical Guide 2012 (in Japanese)	Less than 10 g/dL	10–12 g/dL. Try to keep under 12 g/dL. Do not exceed 13 g/dL
KDIGO Guidelines 2012	Less than 10 g/dL	10–11.5 g/dL. Do not exceed 13 g/dL

### Initial dose

In adults, 30 µg of darbepoetin alfa is administered once every 2 weeks, subcutaneously or intravenously.

### Maintenance dose

30–120 µg of darbepoetin alfa will be administered once every 2 weeks subcutaneously or intravenously as a maintenance dose. If the improvement is attained with the specific dosage by every 2 weeks administration, the dose can be doubled and administered once every 4 weeks. The dose may be adjusted as needed based on the severity of the anemia, age, and other factors; however, the maximum dose administered should be 180 µg per treatment or less.

### Dose adjustment

The dose may be increased or decreased if the Hb concentration or hematocrit level does not increase appropriately, or if the Hb concentration or hematocrit level falls outside the target range consecutively twice during maintenance administration.

### Changes of the administration interval

1. Before extending the administration interval, changes in Hb concentration and hematocrit level are carefully observed. After confirming that the Hb concentration and hematocrit level has remained stable at a specific dose, the dose may be increased and the interval is extended. After any changes in dosage schedule, the fluctuations in Hb concentration and hematocrit levels should be monitored and dosing should be adjusted.
2. If the Hb concentration or hematocrit level does not reach the target range even by the administration of 180 µg per treatment, the dosage will be reduced by half and administration interval is changed from once every 2 weeks to once per week, or from once every 4 weeks to once every 2 weeks.

### Termination criteria

If any of the events below occur, events are recorded on the case report form and on termination report form, and submitted to the Translational Research Informatics Center.

1. Patient’s death.
2. A patient cannot be contacted for follow-up.
3. A patient requests to terminate the participation of the trial. In this case, the patient’s information before termination can be included in this trial.
4. A patient withdraws his/her consent. In this case, the patient’s information after registration cannot be included in this trial.
5. A patient whom the investigator deems appropriate to terminate.

### Observation periods and parameters

The following parameters are observed or examined from the initiation of the trial until the end or termination of this trial. The results are recorded on online case report forms. Case registration is performed by the registered physicians of each institute.

At registration, patient information, eligibility, and blood tests are examined. Before the initiation of the study, primary disease, past history, complications, smoking history, height, pulse wave velocity (PWV), 12-lead electrocardiogram, and chest radiography are examined and recorded. During the observation period from the initiation of the therapy until 96 weeks after the administration, the administration status of darbepoetin alfa, iron, and other drugs, body weight, blood pressure, blood test, HbA1c, urinalysis, administration of red-cell transfusions, and other factors that may affect anemia, renal/CVD events, adverse events, and outcome are examined and recorded on the week of 2, 4, 6, 8, 10, 12, 16, 24, 36, 48, 60, 72, 84, and 96. Complete blood count, serum creatinine, albumin, iron, ferritin, and total iron-binding capacity (TIBC) are measured at the beginning of the study and on the week of 2, 4, 6, 8, 10, 12, 16, 24, 36, 48, 60, 72, 84, and 96. In addition, high sensitivity CRP, folic acid, vitamin B12, NT-pro BNP, iron, ferritin, and TIBC are measured at the clinical laboratory company (SRL, Tokyo, Japan). 96 weeks after the last patient is administered, general survey of outcomes of all patients will be performed including the survival, fatal and non-fetal CVD events, renal outcomes, and adverse events.

### Definition of events

This trial defined the events as the following deteriorations in renal function and CVD. A deterioration in renal function is defined as the initiation of maintenance dialysis, kidney transplantation, 50% decrease in eGFR, or eGFR of 6 mL/min/1.73 m^2^ or less. A fatal CVD event is defined as death due to myocardial infarction, congestive heart failure, arrhythmia, cerebrovascular diseases, aortic dissection, other forms of cardiovascular diseases, ischemia in major organs, and sudden death. A nonfatal CVD event is defined as hospitalization due to myocardial infarction, angina pectoris, ischemic heart disease requiring invasive treatment, congestive heart failure, severe arrhythmia, atrial fibrillation, atrial flutter, aortic dissection, and ischemia of major organs.

### Sample size and power

The target number of cases to be registered for this trial enough to evaluate ESA hyporesponsive patients was set at 2000. This number was calculated after considering the number of data necessary for each assessment and analysis. In the TREAT trail [9], the 2 year incidence rate for renal and CVD events in the darbepoetin alfa group was from 23 to 24%. Thus, assuming that 2000 cases were enrolled and observed over 2 years and an event incidence is 13.1 per 100 people, events would occur in 480–586 cases at a 95% confidence interval. This was considered a sufficient number of events for the analyses.

### ERI

For the assessment ESA responsiveness, we will apply the following formulae.

1. ERI-1A = $$\frac{{{\text{The \; doses\; of\; darbepoetin\; alfa\; on\; the\; week\; of }}\;12\; (\upmu {\text{g}})}}{{{\text{The\; concentration\; of\; Hb}}\; \left( {{\text{g}}/{\text{dL}}} \right) \times {\text{body \; weight }}\;({\text{kg}})}}.$$
2. ERI-1B = $$\frac{{{\text{The\; doses\; of\; darbepoetin \; alfa \; on\; the week of }}\;12\; (\upmu {\text{g}})}}{{{\text{The\; concentration\; of \; Hb }}\;\left( {{\text{g}}/{\text{dL}}} \right) }}.$$
3. ERI-2A = $$\frac{{{\text{The \; total \; doses\; of \; darbepoetin \; alfa\; until\; the \; week \; of }}12\; (\upmu {\text{g}})}}{{\Delta {\text{Hb}}\; \left( {{\text{g}}/{\text{dL}}} \right) \times {\text{body weight}}\; ({\text{kg}})}}.$$
4. ERI-2B = $$\frac{{{\text{The\; total \; doses\; of\; darbepoetin \; alfa \; until\; the \; week\; of }}\;12 \;(\upmu {\text{g}})}}{{\Delta {\text{Hb }}\left( {{\text{g}}/{\text{dL}}} \right) }}.$$

∆Hb (g/dL) is calculated as follows: ∆Hb (g/dL) = Hb (g/dL) at 12 week − Hb (g/dL) before administration.

In addition to the application of the data at 12 week, we will apply the data at 16 and 24 week to the formulae above and evaluate the correlation between these ERIs and CVD events.

### Statistical analysis

A proportional hazard model with covariates will be applied to examine factors that influence event incidence of renal and CVD. The model will be examined by adding and subtracting covariates as needed. The presence or absence of complicating diabetes, arteriosclerosis, and chronic inflammation is considered stratification factors. These analyses will be applied to investigate formulas to predict event incidence from covariates.

The cut-off values for each ERI will be increased gradually from minimum to maximum to create time-dependent ROC curves of survival time data until occurring of the events, allowing comparison of the areas under the curves.

When cut-off values for an ERI are determined, two groups will be created based on the cut-off value, and then, a cumulative survival curve is estimated using Kaplan–Meier method, and yearly incidence event rates and 95% confidence intervals will be estimated. Exploratory analysis for the ERI cut-off values will also be examined using cross-validation methods. Patients’ data are expressed as mean ± standard deviation.

## Results

We will investigate the conditions of hyporesponsive patients and factors related to hyporesponsiveness to darbepoetin alfa. Using darbepoetin alfa, an ESA commonly used to treat anemia in CKD patients, we aim to gather a wide range of clinical information of ESA therapy to investigate the status of patients who are hyporesponsive to ESA and factors that are related to low responsiveness. Another objective was to examine relationship between ESA hyporesponsiveness and occurring renal and CVD events based on the clinical situation in Japan to explore an ESA response index.

We planned to enroll 2000 patients. In Dec, 2015, the total number of patients enrolled was 843 from 162 institutes, and thus, the registration deadline was extended to Sep, 2016. As of Oct 17, 2016, we have registered 1977 patients. Data of 1911 patients were eligible and baseline patients’ characteristics are presented in Table 2.

**Table 2:** Baseline characteristics of the patients

Age (years)	70.0 ± 12.1
Number of females (%)	789 (41.3%)
Cause of CKD (%)	
Chronic glomerulonephritis	418 (21.9%)
Nephrosclerosis	423 (22.1%)
Polycystic kidney disease	97 (5.1%)
Chronic pyelonephritis	3 (0.2%)
Diabetic nephropathy	515 (26.9%)
Lupus nephritis	14 (0.7%)
Others	330 (17.3%)
Unknown	111 (5.8%)
Serum Cre (mg/dL)	2.9 ± 1.4
eGFR mL/min/1.73 m^2^	20.3 ± 10.0
Urinary protein (mg/g Cre)	3.5 ± 9.7
Baseline hemoglobin (g/dL)	9.8 ± 1.2
Fe (μg/dL)	71.0 ± 28.4
TIBC (μg/dL)	267.8 ± 90.6
Ferritin (ng/mL)	159.0 ± 151.8

## Discussion

Even though targeting higher hemoglobin levels in non-dialysis CKD patients with ESA has been shown to preserve renal functions [5, 6], the outcome of renal and CVD events is still controversial [8, 9, 13]. It is unclear whether the pathology causing the hyporesponsiveness itself leads to poor prognosis or the administration of high-dose ESA needed to achieve target Hb levels in hyporesponsive patients worsen prognosis [10, 11]. ESA hyporesponsiveness should be defined by investigating relationships with prognosis in prospective trials, but the evidence for this does not yet exist. If patient groups that exhibit ESA responsiveness could be properly diagnosed, the characteristics that contribute to poor prognosis could be determined and more effective treatment guidelines could be created, which would like to improve prognoses among these patients.

In addition, the evidence available to date has extensively been derived from Western countries, and there are no large data sources on Japanese subjects. As Western and Japanese CKD patients are considered to have different characteristics and lower CVD events than Western CKD patients [6, 14], and they actually are under different clinical practice [15], examining and defining the ESA hyporesponsiveness in Japanese non-dialysis CKD patients are important to establish the characteristics of ESA hyporesponsiveness and provide appropriate therapy for ESA hyporesponsive patients in Japan.

This trial would establish evidence for ESA hyporesponsiveness and contribute to improve therapies for renal anemia.
